# Simultaneous Differential Detection of H5, H7, H9 and Nine NA Subtypes of Avian Influenza Viruses via a GeXP Assay

**DOI:** 10.3390/microorganisms12010143

**Published:** 2024-01-11

**Authors:** Sisi Luo, Zhixun Xie, Meng Li, Dan Li, Minxiu Zhang, Zhihua Ruan, Liji Xie, Sheng Wang, Qing Fan, Yanfang Zhang, Jiaoling Huang, Tingting Zeng

**Affiliations:** Guangxi Key Laboratory of Veterinary Biotechnology, Key Laboratory of China (Guangxi)-ASEANCross-Border Animal Disease Prevention and Control, Ministry of Agriculture and Rural Affairs of China, Guangxi Veterinary Research Institute, Nanning 530001, China; 2004-luosisi@163.com (S.L.); mengli4836@163.com (M.L.); lidan8217@163.com (D.L.); zhminxiu2010@163.com (M.Z.); ruanzhihua2020@163.com (Z.R.); xie3120371@163.com (L.X.); wangsheng1021@126.com (S.W.); fanqing1224@126.com (Q.F.); zhangyanfang409@126.com (Y.Z.); huangjiaoling728@126.com (J.H.); tingtingzeng1986@163.com (T.Z.)

**Keywords:** H5 subtype, H7 subtype, H9 subtype, nine NA subtypes, avian influenza, GeXP assay

## Abstract

H5, H7 and H9 are the most important subtypes of avian influenza viruses (AIVs), and nine neuraminidase (NA) subtypes (N1–N9) of AIVs have been identified in poultry. A method that can simultaneously detect H5, H7, H9 and the nine NA subtypes of AIVs would save time and effort. In this study, 13 pairs of primers, including 12 pairs of subtype-specific primers for detecting particular subtypes (H5, H7, H9 and N1–N9) and one pair of universal primers for detecting all subtypes of AIVs, were designed and screened. The 13 pairs of primers were mixed in the same reaction, and the 13 target genes were simultaneously detected. A GeXP assay using all 13 pairs of primers to simultaneously detect H5, H7, H9 and the nine NA subtypes of AIVs was developed. The GeXP assay showed specific binding to the corresponding target genes for singlet and multiplex templates, and no cross-reactivity was observed between AIV subtypes and other related avian pathogens. Detection was observed even when only 10^2^ copies of the 13 target genes were present. This study provides a high-throughput, rapid and labor-saving GeXP assay for the simultaneous rapid identification of three HA subtypes (H5, H7 and N9) and nine NA subtypes (N1–N9) of AIVs.

## 1. Introduction

Influenza can occur in pandemics and localized outbreaks. Avian influenza viruses (AIVs) belong to the influenza A type in the family *Orthomyxoviridae.* The hemagglutinin (HA) and neuraminidase (NA) proteins are often considered the most important viral antigens, and eighteen HA subtypes (H1–H18) and eleven NA subtypes (N1–N11) have been identified based on differences in the HA and NA antigens; sixteen HA subtypes (H1–H16) and nine NA subtypes (N1–N9) have been recognized and found in poultry and wild birds, while two additional HA and NA subtypes, H17N10 and H18N11, have been identified in bats [1,2]. AIVs, especially subtypes H5, H7 and H9, have contributed to enormous economic losses and pose a potential threat to global human public health. H5 and H7 are highly pathogenic and cause serious illness and death in domestic poultry and humans [3,4]. Infection with influenza A virus subtypes H5, H7 and H9 causes several respiratory diseases in humans. H5, H7 and H9 are the most important subtypes in poultry. In 1997, a human fatality occurred due to H5N1-subtype AIV infection in Hong Kong, and an outbreak of the disease caused panic and serious economic losses in the poultry industry [5]. In recent years, new H5 subtype combinations, H5N6 and H5N8, have emerged and caused a pandemic in poultry, even causing human infections [6,7]. In 2013, a fatal case of H7N9 infection occurred in the Yangtze River Delta region of China, and this virus spread to most parts of the country, causing five waves of infection [8]. Currently, H7N9 is controlled by vaccination, but it still poses a serious threat to public health. H9 is commonly isolated and identified in poultry and provides internal genes to H5 and H7 viruses that can infect humans, such as H7N9 and H5N6 [9,10]. The H9 subtype has been detected in domestic fowl and wild birds in various regions and is considered one of the most likely causes of new influenza pandemics in humans. In recent years, viruses in which H5 and H7 are combined with different NA subtypes, such as H5N2, H5N6, H5N8, H7N2, H7N7 and H7N9, have emerged continuously [11]. The H9 subtype of AIV has been associated with every one of the known nine NA subtypes described and mainly combines with N2 [12]. Rapid differential diagnosis of avian influenza viruses is of paramount importance to the poultry industry and public health. 

Viral culture is still used as a standard reference method for routine surveillance of influenza viruses. However, the sample must first be cultured by inoculating cells or chicken embryos, and 3–5 days are needed for virus proliferation; then, the viral culture medium is collected and tested by hemagglutination inhibition and neuraminidase inhibition to determine the HA and NA subtypes, which requires a series of specific positive serums. This process is time-consuming and laborious. The use of viral culture, an essential tool in the epidemiological surveillance and diagnosis of influenza viruses, has been relegated by the use of more sensitive and affordable molecular techniques [13]. For example, in molecular assays, especially in multiplex formats, a multiplex reverse transcriptase polymerase chain reaction (conventional RT–PCR) was developed to simultaneously differentiate the avian H5, H7 and H9 subtypes [14]. Accurate, rapid and triplex real-time fluorescent quantitative RT-PCR assays were developed for the simultaneous detection of the AIV subtypes H5, H7 and H9 [15]. Three loop-mediated isothermal amplification (LAMP) methods were developed to detect the presence of AIVs and discriminate between the H5 and H9 subtypes, which need three different reaction systems [16]. However, all of these methods can simultaneously detect only two to four pathogens (target genes) and cannot realize high-throughput detection. Simultaneous detection of HA and NA subtypes of AIVs has rarely been reported, and no previous studies have conducted simultaneous detection of H5, H7, H9 and the nine NA subtypes.

Rapid differentiation of the important AIV subtypes H5, H7 and H9 and the nine NA subtypes from other pathogens may require many reactions. There are many subtypes of AIVs, and rapid differential diagnosis is difficult. Detection methods for the simultaneous identification of the H5, H7 and H9 subtypes and nine NA subtypes are very important and rare. The GenomeLab Gene Expression Profiler genetic analysis system (GeXP) is a new high-throughput detection platform that uses a novel, modified RT-PCR process that converts multiplexed RT-PCR with several pairs of primers to a primer-pair process using universal primers, followed by fluorescence capillary electrophoresis separation based on the size of the amplified products. The amplified fragments are separated by capillary electrophoresis to generate visual fluorescence signal peaks to simultaneously detect and identify multiplex pathogens or genes. Our study aimed to establish a GeXP assay for the simultaneous identification of the H5, H7 and H9 subtypes and nine NA subtypes of AIVs by using GeXP technology; we simultaneously detected 13 target genes in one tube, indicating that this is a time-saving, simple, rapid and high-throughput process. We previously obtained partial data from a GeXP assay for simultaneous differential detection of N1–N9-subtype AIVs, and that assay was used for differential diagnosis of NA-subtype AIVs [17]. This study focused on the simultaneous detection of H5-, H7-, H9- and nine candidate NA-subtype AIVs and decreased the number of reactions from 13 to 1. This method could be used not only for the simultaneous detection of the 12 specific subtypes but also for determining the combinations of H5, H7 and H9 with the NA subtypes in a single reaction tube, which is especially important for the prevention and control of AIV infection.

## 2. Materials and Methods

### 2.1. Design of High-Performance, Gene-Specific Primers for a GeXP Multiplex

To design the gene-specific primers, which included H5, H7, H9 and N1–N9 AIV subtype-specific primers and AIV subtype-universal primers, the HA gene sequences of the H5, H7 and H9 subtypes and other HA subtypes, the NA gene sequences of the N1–N9 subtypes and the M gene sequences of all subtypes were obtained from the Influenza Virus database at the National Center for Biotechnology Information (NCBI) (https://www.ncbi.nlm.nih.gov/genomes/FLU; accessed on 25 November 2023). First, the conserved regions and sequences of the HA and NA subtypes of AIVs were identified via MegAlign in DNASTAR-Lasergene 8.0 software. Second, specific primers were designed for the conserved sequences using Primer Premier 5.0 software, in which the annealing temperature, mismatches and dimers of primers were evaluated, and some candidate primers were preliminarily selected. Finally, the primers used were examined in silico via BLAST comparisons online (https://blast.ncbi.nlm.nih.gov/Blast.cgi; accessed on 25 November 2023) to determine the specificity of crossing with other subtypes of AIVs and pathogens in vitro. After comparison and verification of the primers, two to three pairs of candidate primers were screened for each target gene. Primers’ amplicons 100–350 bp in length were designed (without universal tags). The GeXP universal tag (the underlined sequence in Table 1) was added to the 5′ end of the gene-specific forward and reverse primers, and the complete primers obtained were referred to as chimeric primers. The universal tag sequence was added to each of the gene-specific primer sequences to obtain the final primer sequence. The designed fragment size was the specific gene size plus 37 bp for the universal tags; thus, amplicons between 137 and 387 bp in size were designed with universal tags. Amplicons were designed such that each fragment was no less than 5 nucleotides away from its nearest neighbor, which allowed for variation in migration to meet the minimum peak separation distance of 3 nucleotides. All primers were synthesized and purified by Invitrogen (Guangzhou, China).

### 2.2. Viral DNA/RNA Nucleic Acid Extraction

Reference strains and field isolates of the H5, H7, H9 and N1–N9 AIV subtypes, other avian pathogens (including infectious Newcastle disease virus (NDV), infectious bronchitis virus (IBV), laryngotracheitis virus (ILTV), avian reovirus (ARV) and fowl adenovirus virus serotype four (FAdV4)) and human influenza B virus were used in this study (see Table S1 in the supplemental information online). Viral RNA/DNA was extracted from 200 µL of virus stock using a Viral DNA/RNA Extraction Kit (TransGen, Beijing, China) according to the manufacturer’s instructions. The extracted RNA was reverse transcribed to synthesize cDNA using the cDNA Synthesis Kit (Takara, Dalian, China), AIVs were reverse transcribed using a 12 bp primer (5′-agcgaaagcagg-3′), non-AIV viruses were reverse transcribed using random primers in the kit, and the cDNA and DNA were stored at −20 °C.

### 2.3. Reaction Procedures and Conditions of the GeXP Assay

The GeXP assay reaction system had a total volume of 20 µL, including 4 µL of 5 × PCR buffer (GenomeLab GeXP Start Kit, Beckman Coulter, Brea, CA, USA; universal forward and reverse primers were formulated in 5 × PCR buffer; fluorescently labeled universal forward primer: 5′-Cy5-AGGTGACACTATAGAATA-3′; universal reverse primer: 5′-GTACGACTCACTATAGGGA-3′), 4 µL of 25 mM MgCl_2_, 1 µL of 2.5 U/µL JumpStart Taq DNA polymerase (Sigma, St. Louis, MO, USA), 2 µL of the 13-primer-pair mixture (200 nM each primer, Table 1) and 1–2 µL of cDNA, with nuclease-free water added to 20 µL. The reaction system was placed in a PCR amplification instrument (Bio-Rad, Hercules, CA, USA). The tubes were incubated at 95 °C for 5 min, followed by three steps of amplification according to the temperature switch PCR (TSP) strategy [18]: step 1, 10 cycles of 30 s at 94 °C, 30 s at 55 °C and 30 s at 72 °C; step 2, 10 cycles of 30 s at 94 °C, 30 s at 62 °C and 30 s at 72 °C; step 3, 20 cycles of 30 s at 94 °C, 30 s at 50 °C and 30 s at 72 °C; 5 min at 72 °C and held at 12 °C in a thermal cycler (Bio-Rad). After amplification, 2 µL of PCR product was added to 37.75 µL of sample loading solution along with 0.25 µL of DNA standard-400 (including in the GenomeLab GeXP Start Kit). The PCR products (the fluorescence-labeled amplicons) were separated and analyzed with a GeXP instrument (Beckman Coulter). After approximately 50 min, the PCR products were separated through capillary electrophoresis, and the results were presented as separated peaks on the electropherogram, which were identified according to the respective sizes. The dye signal strength of each peak was measured as the A.U. of optical fluorescence and defined as the fluorescence signal minus the background. The data were analyzed using GeXP system. The horizontal coordinate is the size of the amplified fragment, and the vertical coordinate is the fluorescence signal value.

### 2.4. Validation of a Single Primer and Preliminary Evaluation of Multiple Primers

The primers used for each target gene were first validated with a single pair of primers and cDNA to validate whether they could be amplified efficiently, and DNA/cDNA from other related pathogens was detected as a template to validate primer specificity according to the GeXP procedures. Only primers with good amplification efficiency and strong specificity can be candidate multiplex GeXP primers. Thirteen pairs of primers were selected and mixed as multiplex primers for the GeXP assay to determine whether the corresponding target gene could be amplified and whether cross-reaction occurred between primers.

### 2.5. Evaluation of the Specificity of the GeXP Assay

The GeXP assay was established using 13 screened and verified pairs of primers. To assess the specificity of the GeXP assay and determine whether the GeXP assay could correctly identify H5-, H7-, H9- and nine NA-subtype AIVs and whether any cross-reactivity occurred with other related pathogens, reference strains and field isolates of the H5N1, H5N6, H7N2, H7N7, H9N2 and N1–N9 subtypes (including H3N2, H4N2, H6N2, H2N3, H10N3, H8N4, H12N5, H3N6, H4N6, H6N6, H3N8, H4N8, H5N9 and H7N9) and other AIVs; human influenza B viruses; and other related avian pathogens (NDV, IBV, ILTV, ARV and FAdV4) listed in Table S1 were individually detected using cDNA/DNA from the GeXP assay. Multiplex templates were also detected using the GeXP assay.

### 2.6. Evaluation of the Sensitivity of the GeXP Assay

The target fragments of the 13 pairs of primers (Table 1) were amplified, cloned and inserted into the pGEM-T vector to obtain 13 recombinant plasmids, which were verified by sequencing. The 13 recombinant plasmids were mixed in equal amounts and serially diluted with 10^6^–10 copies. Based on the sensitivity of GeXP, we expected to detect at least 10^3^ copies. A total of 10^3^–10 copies of these recombinant plasmids, including the 13 target fragments, were evaluated via the GeXP assay for sensitivity. The concentrations of the 13 recombinant plasmids were adjusted according to the test results, and sensitivity evaluation was performed via the GeXP assay.

### 2.7. Application for Detecting Clinical Samples

A total of 150 swab samples were randomly collected from the cloacae and larynges of healthy chickens, geese and ducks from five live bird markets (LBMs) in 2022 in Guangxi Province, China. The swabs were then suspended in 1 mL of storage medium containing antibiotics at 4 °C until arrival at the laboratory. The swab sample treatment method was previously described [19]. Viral RNA was extracted from swab samples, and cDNA was reverse transcribed according to previously described protocols (Section 2.2 in this paper). The cDNA of different tissue samples of three H5N2-infected SPF chickens was donated by the University of Connecticut, and the cDNA of different tissue samples of five H7N9-infected SPF chickens was donated by China Agricultural University. Swab and tissue samples were simultaneously examined via the GeXP assay and real-time PCR (using the same primers as the GeXP assay and TB Green Premix Ex Taq from Takara). The HA and NA genes of the positive samples were amplified, cloned using previously described primers [20] and sequenced by Invitrogen (Guangzhou, China).

## 3. Results

### 3.1. Screening Results for the 13 Pairs of Primers

First, to evaluate the single pair of primers for each target, each pair of primers was tested based on its corresponding singlet template; other subtypes of AIVs, other related avian pathogens and human influenza B virus were subsequently used to test the specificity of the single pair of primers. Then, each target was screened with a pair of candidate primers, which were combined without overlapping amplification fragment sizes. Second, to evaluate the multiplex primers used for singlet and multiplex templates, 13 pairs of target primers were mixed together, and singlet and multiplex templates of H5, H7, H9 and N1–N9 were detected to determine whether the primer combinations could amplify the corresponding targets. Other subtypes of AIVs, other related avian pathogens and human influenza B virus were subsequently used to test the specificity of the multiplex primers. If the amplification efficiency of the primers was low or if no target peak was observed, the primers were replaced until the optimal 13 pairs of primers were identified. The 13 pairs of primers that were ultimately selected are shown in Table 1, and these were used to develop the GeXP assay. In this study, fragments of the expected sizes were amplified for the H5, H7, H9 and N1–N9 subtypes AIVs: H5, 227 to 231 bp; H7, 140 to 145 bp; H9, 329 to 334 bp; N1, 244 to 249 bp; N2, 279 to 285 bp; N3, 215 to 221 bp; N4, 149 to 155 bp; N5, 295 to 301 bp; N6, 236 to 241 bp; N7, 192 to 198 bp; N8, 173 to 178 bp; N9, 205 to 211 bp; and AIV, 158 to 164 bp.

### 3.2. Evaluation of the Specificity of the GeXP Assay

**Figure 1:**
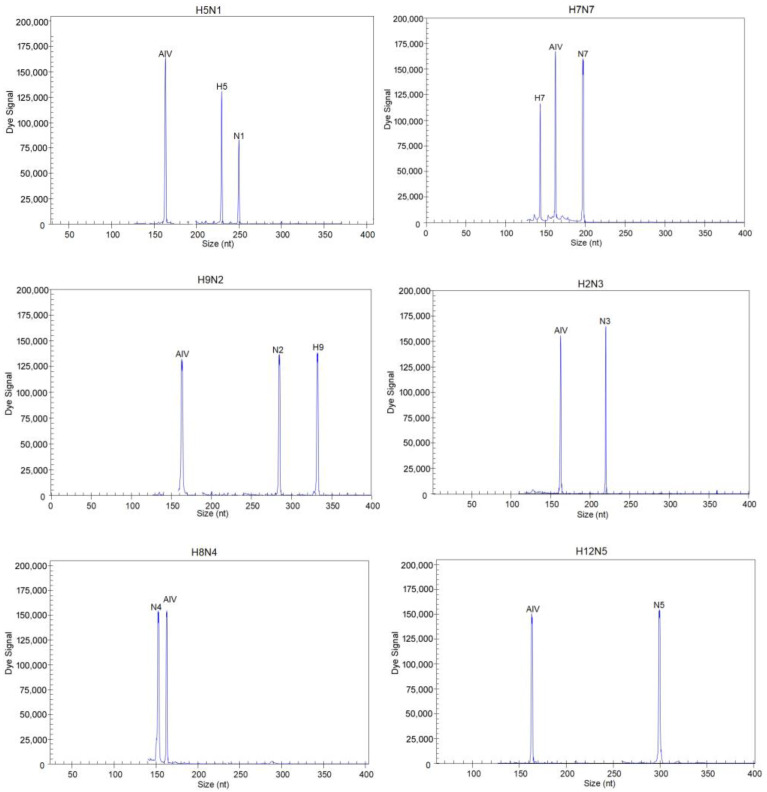

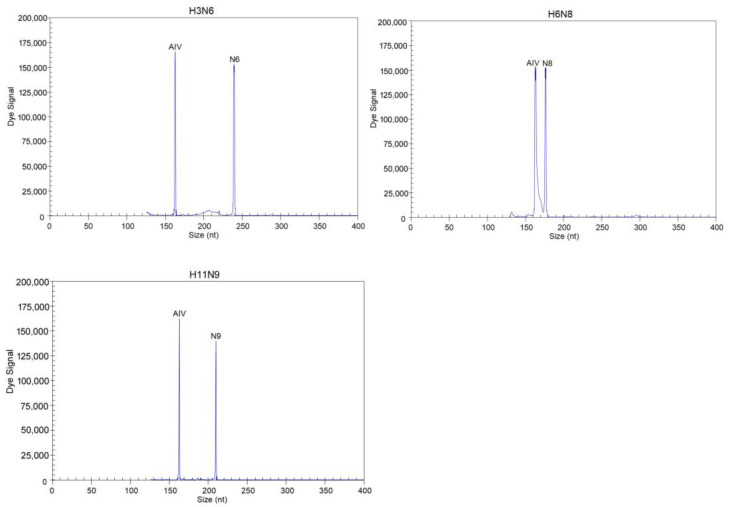
Specificity of the GeXP assay for different AIV subtype templates. The *Y*-axis indicates the dye signal in arbitrary units, and the *X*-axis indicates the actual PCR product size.

Specific results were obtained for different subtypes of AIVs and other pathogens using the GeXP assay (Table S1). Single templates of the H5, H7, H9 and nine NA subtypes were detected using the GeXP assay, and the corresponding target peaks were amplified (Figure 1). For the H5, H7 and H9 subtypes, three target peaks were generated; for example, H5N1 produced H5- and N1-subtype-specific peaks and the universal AIV detection peak; H7N7 produced H7- and N7-subtype-specific peaks and the universal AIV detection peak; and H9N2 produced H9- and N2-subtype-specific peaks and the universal AIV detection peak. The other subtypes exhibited two peaks: an NA-subtype-specific peak and a universal detection peak. When multiple templates were mixed, the corresponding target peaks could be detected. Each primer showed specific amplification peaks, and in the detection of other related pathogens, no amplification peaks were observed, demonstrating the superior specificity of the GeXP assay.

### 3.3. Evaluation of the Sensitivity of the GeXP Assay

**Figure 2:**
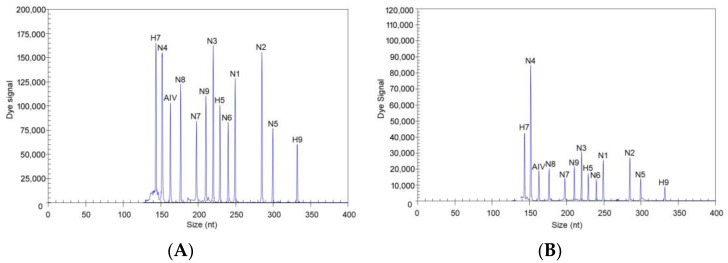
Sensitivity of the GeXP assay for the simultaneous detection of 13 target genes. All 13 premixed viral targets were detected. (**A**) 10^3^ copies; (**B**) 10^2^ copies.

To evaluate the sensitivity of the GeXP assay, 13 recombinant plasmids, each containing a target fragment, were mixed, diluted and detected using the GeXP assay, wherein 13 target genes were simultaneously detected in one tube. The results showed that when all 13 recombinant plasmids were present at 10^3^ copies, the target peaks were all observed (Figure 2A). When the concentration of the 13 plasmids was diluted so that 10^2^ copies of each plasmid were present, all the plasmids were still detected via the GeXP assay (Figure 2B). The reaction at each template concentration was repeated three times, and similar results were obtained (CV ≤ 7.23% for each concentration).

### 3.4. Detection of AIVs in Clinical Samples Using the GeXP Assay

All the swab samples were tested using the GeXP assay, and the samples were confirmed by real-time PCR and sequencing. The positive and negative results obtained using the various methods and the agreement among the GeXP assay, real-time RT-PCR method and sequencing results are presented in Table 2. The H9N2, HxN2 and HxN6 subtypes of AIV were the most common in the positive samples. The GeXP assay yielded 100% specificity, in contrast to the conventional approaches, and GeXP was superior to the simultaneous detection of multiple pathogens. The cDNA of different tissue samples of three H5N2-infected SPF chickens donated by the University of Connecticut and the cDNA of different tissue samples of five H7N9-infected SPF chickens donated by China Agricultural University were detected using the GeXP assay, real-time PCR and sequencing, and the results indicated that the GeXP assay could detect H5, H7 and the corresponding NA subtypes (Table 2).

## 4. Discussion

Globalization and industrialization over the past few decades have contributed to the emergence of novel influenza viruses that threaten animal and human health. Avian influenza is an important pathogen that continually threatens both human and animal health. H5- and H7-subtype AIVs have caused severe problems in the global poultry industry and pose severe threats to public health [21]. Highly pathogenic H5N1 AIVs continue to circulate in avian populations, resulting in sporadic infections in humans with a high mortality rate [22]. The H7-subtype AIV HA gene has been found in combination with all nine NA-subtype genes [23]. During the past few years, infections in poultry and humans with H7 subtypes have increased markedly. H9-subtype AIV, which exhibits low pathogenicity, has been reported to be capable of infecting humans, and the World Health Organization has warned that the H9N2 subtype could trigger a global influenza outbreak in humans [24,25]. H9 is found mainly in N2, and H7N9 and H9N2 coinfection has been shown to produce H9N9 [26]. The detection of H5, H7 and H9 is essential. In this study, using the developed GeXP assay, H5-, H7- and H9-subtype AIVs were simultaneously identified, and nine NA subtypes were also identified; one pair of primers was used for the detection and validation of all AIV subtypes.

Subtyping by conventional RT-PCR or real-time RT-PCR is a common method that can be performed directly on nucleic acids from clinical specimens. However, these approaches are often used to detect single pathogens, and even the detection of multiple pathogens is often limited to 2–3 species, as the detection results are disrupted by competitive amplification between primers or interference by different fluorophores. Conventional RT-PCR and real-time RT-PCR can thus be difficult to develop as high-throughput tests and have limited benefits in terms of the number of pathogens that can be simultaneously detected. GeXP can integrate reverse transcription PCR (RT–PCR) and labeled amplified products in multiplex PCR assays. GeXP technology can enable more effective detection of multiple pathogens, wherein 30 target genes can be detected simultaneously, saving reaction time and accelerating detection. The unique feature of GeXP-based multiplex PCR is that amplification by multiplex primers was converted into amplification by a pair of universal primers. At the beginning of the reaction, chimeric primers were first used to amplify the target gene to produce PCR products with GeXP universal primers. Amplification by the universal primers is rapid since universal primers are present at significantly higher concentrations than gene-specific primers within the PCRs; this gradually led to the transformation of a procedure based on multiple chimeric primers into a procedure involving a single pair of GeXP universal primers. Thus, we used a temperature switch PCR(TSP) procedure referring to a previous study [18]: step 1 was carried out using gene-specific sequences of chimeric forward and reverse primers, step 2 was carried out mainly using chimeric forward and reverse primers and step 3 was predominantly carried out using universal forward and reverse primers. Compared with conventional PCR, real-time PCR and LAMP, the GeXP assay can detect many more target genes simultaneously, and competitive amplification and interference between primers are greatly reduced via this approach. A major advantage of GeXP is that this technique can provide a broad detection range for the identification of pathogens that previously needed multiple laboratory subtype assays. Moreover, GeXP provides more intuitive and detailed data than conventional molecular subtyping tests.

The primers used were designed based on the HA and NA gene sequences of different AIV subtypes, allowing differentiation of the HA and NA subtypes of AIVs, respectively. The M gene is the conserved gene of all AIV subtypes and was used to detect all AIV subtypes. Thirteen pairs of gene-specific primers were selected for analysis as follows: (1) After the 13 pairs of primers were mixed, each pair of primers in the mixture could amplify its own target with the corresponding template, and cross-amplification of the remaining 12 target genes and other related pathogens did not occur. (2) Primers had little influence on each other’s amplification efficiency, and the amplification efficiency was similar to that of a single template with a single pair of primers. (3) The amplicon lengths obtained with the 13 pairs of primers ranged from 137 to 387 bp, and the amplification size of each target fragment was greater than 5 bp. The whole GeXP process for eight samples was completed in approximately 5 h (1.5 h for nucleic acid extraction and reverse transcription, 2.5 h for PCR, and eight samples/1 h for electrophoresis). The time used was the same as that used for conventional RT-PCR; real-time RT-PCR may take 2.5 h to detect the same number of samples, but the GeXP assay can be used to test more targets in a short period. If real-time RT-PCR is used to detect the same targets, additional time may be needed. The cost of the GeXP assay for the simultaneous detection of 12 subtypes is approximately USD 7 per test, and the cost of detection is comparable to that of real-time RT-PCR. In addition, two 96-well plates can be placed in parallel in a GeXP machine at the same time to further increase sample throughput.

Among the clinical samples, the N3-, N4-, N5-, N7- and N9-subtype AIVs were not detected. These NA subtypes were less prevalent. This GeXP method has been developed based on local AIV strains, allowing simultaneous and specific detection of H5, H7, H9 and nine NA subtypes of AIVs, and can be used as an efficient and novel method for the prevention and control of AIVs. In conclusion, in this study, we developed a GeXP assay that serves as a specific, sensitive, rapid, high-throughput tool for the simultaneous detection of the H5, H7, H9 and N1–N9 subtypes of AIVs.

## Figures and Tables

**Table 1:** Information for the 13 pairs of primers used in the GeXP assay.

Virus	Forward Primer (5′-3′)	Reverse Primer (5′-3′)	Gene	GenBank Accession Number
AIV	AGGTGACACTATAGAATA AGGCTCTCATGGAGTGGCTA	GTACGACTCACTATAGGGA TGGACAAAGCGTCTACGCTG	M	OQ830456.1
AIV-H5	AGGTGACACTATAGAATA ATACACCCTCTCACCATCGG	GTACGACTCACTATAGGGA TTGCTGTGGTGGTACCCATA	HA	OQ546777.1
AIV-H7	AGGTGACACTATAGAATA AGAATACAGATTGACCCAGT**S**AA	GTACGACTCACTATAGGGA CCCATTGCAATGGC**H**AGAAG	HA	MK453329.1
AIV-H9	AGGTGACACTATAGAATA ATGGCAA**Y**CCTTC**Y**TGTGA	GTACGACTCACTATAGGGA TTGTGTATTGGGCGTC**Y**TG	HA	OR528241.1
AIV-N1	AGGTGACACTATAGAATA GGTGTTTGGATCGG**R**AGAAC	GTACGACTCACTATAGGGA TCAACCCAGAA**R**CAAGGTC	NA	OP373692.1
AIV-N2	AGGTGACACTATAGAATA TTGGGTGTTCCGTTTCA	GTACGACTCACTATAGGGA CCATCCGTCATTACTAC	NA	OQ954751.1
AIV-N3	AGGTGACACTATAGAATA TTCCCAATAGGAACAGC**Y**CCAGT	GTACGACTCACTATAGGGA TTCTCCATGATTT**R**ATGGAGTC	NA	MK978938.1
AIV-N4	AGGTGACACTATAGAATA CAGA**Y**AAGGA**Y**TCAAATGGTGT	GTACGACTCACTATAGGGA CATGGTACAGTGCAATTCCT	NA	MT421389.1
AIV-N5	AGGTGACACTATAGAATA GTGAGGTCATGGAGAAAGCA	GTACGACTCACTATAGGGA TGG**Y**CTATTCATTCC**R**TTCCA	NA	LC339605.1
AIV-N6	AGGTGACACTATAGAATA CACTATAGATCC**Y**GA**R**ATGATGACC	GTACGACTCACTATAGGGA GGAGTCTTTGCTAAT**W**GTCCTTCCA	NA	MT375537.1
AIV-N7	AGGTGACACTATAGAATA GACAG**R**AC**W**GCTTTCAGAGG	GTACGACTCACTATAGGGA GTTGCGTTGTCATTATTTCC	NA	MN253549.1
AIV-N8	AGGTGACACTATAGAATA AGGGAATACAATGAAACAGT	GTACGACTCACTATAGGGA TGCAAAACCCTTAGCATCACA	NA	MT421085.1
AIV-N9	AGGTGACACTATAGAATA CGCCCTGATAAGCTGGCCACT	GTACGACTCACTATAGGGA ACAGGCCTTCTGTTGTACCA	NA	KP418553.1
GeXP universal primer	AGGTGACACTATAGAATA	GTACGACTCACTATAGGGA		

The universal primer tag sequences are underlined. The bold type shows degenerate sites. R: A/G; Y: C/T; W: A/T; S: G/C; H: A/T/C.

**Table 2:** Detection results for clinical samples using the GeXP assay, real-time RT-PCR and sequencing.

Type of Chicken Samples	Number of Positive Samples	GeXP Assay	Real-Time RT-PCR		Sequencing	
			Three Methods for H5, H7 and H9 Real-Time RT-PCR	Nine Methods for N1–N9 Real-Time RT-PCR	HA Gene	NA Gene
Swab samples from LBMs	8	H9, N2, AIV	H9	N2	H9	N2
	2	N1, AIV	None ^a^	N1	- ^b^	N1
	9	N2, AIV	None ^a^	N2	- ^b^	N2
	5	N6, AIV	None ^a^	N6	- ^b^	N6
	3	N8, AIV	None ^a^	N8	- ^b^	N8
Tissue samples from challenged SPF chickens	3 ^c^	H5, N2, AIV	H5	N2	H5	N2
	3 ^c^	H5, N2, AIV	H5	N2	H5	N2
	3 ^c^	H5, N2, AIV	H5	N2	H5	N2
	3 ^d^	H7, N9, AIV	H7	N9	H7	N9
	3 ^d^	H7, N9, AIV	H7	N9	H7	N9
	3 ^d^	H7, N9, AIV	H7	N9	H7	N9

“a” indicates no positive results according to the three methods for H5, H7 and H9 real-time RT-PCR. “b” indicates that the non-H5, H7 and H9 subtypes were AIVs, which was not relevant to the comparative detection of the developed GeXP method; therefore, the results are not shown here. “c” indicates heart, spleen and lung samples from the same H5N2-infected SPF chicken. “d” indicates heart, spleen and lung samples from the same H7N9-infected SPF chicken.

## Data Availability

The data presented in this study are available within the article.

## Supplementary Materials

The following supporting information can be downloaded at: https://www.mdpi.com/article/10.3390/microorganisms12010143/s1, Table S1: Information and detection result of strains used in the GeXP assay.
