# Supplementary material for: Simultaneous Differential Detection of H5, H7, H9 and Nine NA Subtypes of Avian Influenza Viruses via a GeXP Assay
Source: Microorganisms. 2024 Jan 11;12(1):143. doi: 10.3390/microorganisms12010143 (PMC10819249; doi:10.3390/microorganisms12010143)
Supplement: Supplementary file 1 [file microorganisms-12-00143-s001.zip › microorganisms-2689885-supplementary.pdf]

Simultaneous Differential Detection of H5, H7, H9 and Nine NA Subtypes of Avian Influenza Viruses via a GeXP Assay

Sisi Luo, Zhixun Xie *, Meng Li, Dan Li, Minxiu Zhang, Zhihua Ruan, Liji Xie, Sheng Wang, Qing Fan, Yanfang Zhang, Jiaoling Huang and Tingting Zeng

Guangxi Key Laboratory of Veterinary Biotechnology, Key Laboratory of China (Guangxi)-ASEANCross-Border Animal Disease Prevention and Control, Ministry of Agriculture and Rural Affairs of China, Guangxi Veterinary Research Institute, Nanning 530001, China; 2004-luosisi@163.com (S.L.); mengli4836@163.com (M.L.); lidan8217@163.com (D.L.); zhminxiu2010@163.com (M.Z.); ruanzhihua2020@163.com (Z.R.); xie3120371@163.com (L.X.); wangsheng1021@126.com (S.W.); fanqing1224@126.com (Q.F.); zhangyanfang409@126.com (Y.Z.); huangjiaoling728@126.com (J.H.); tingtingzeng1986@163.com (T.Z.)

* Correspondence: xiezhixun@126.com

Table S1. Information and detection result of strains used in the GeXP assay.

Num	Pathogens/field samples	Source	AIV	H5	H7	H9	N1	N2	N3	N4	N5	N6	N7	N8	N9
1	A/Duck/Guangxi/1/04 (H5N1)AIV cDNA	GVRI	+	+	-	-	+	-	-	-	-	-	-	-	-
2	A/Chicken/Guangxi/1/04 (H5N1)AIV cDNA	GVRI	+	+	-	-	+	-	-	-	-	-	-	-	-
3	A/Goose/Guangxi/2/04 (H5N1)AIV cDNA	GVRI	+	+	-	-	+	-	-	-	-	-	-	-	-
4	A/Chicken/QT35/98 (H5N9) AIV cDNA	UP	+	+	-	-	-	-	-	-	-	-	-	-	+
5	A/Chicken PA/3979/97 (H7N2) AIV cDNA	UP	+	-	+	-	-	+	-	-	-	-	-	-	-
6	A/Chicken/NY/273874/03 (H7N2) AIV cDNA	UC	+	-	+	-	-	+	-	-	-	-	-	-	-
7	A/Duck/42846/07 (H7N7) AIVcDNA	UP	+	-	+	-	-	-	-	-	-	-	+	-	-
8	A/Chicken/Guangxi/NN1/2017(H7N9) AIV cDNA	GVRI	+	-	+	-	-	-	-	-	-	-	-	-	+
9	A/Chicken/Guangxi/YL1/2017(H7N9) AIV cDNA	GVRI	+	-	+	-	-	-	-	-	-	-	-	-	+
10	A/dove/Guangxi/151B24/2016(H9N2)	GVRI	+	-	-	+	-	+	-	-	-	-	-	-	-

	AIV														
11	A/chicken/Guangxi/178C38/2014(H9N2) AIV	GVRI	+	-	-	+	-	+	-	-	-	-	-	-	-
12	A/dove/Guangxi/408P55/2020(H9N2) AIV	GVRI	+	-	-	+	-	+	-	-	-	-	-	-	-
13	A/chicken/Guangxi/449C11/2021(H9N2) AIV	GVRI	+	-	-	+	-	+	-	-	-	-	-	-	-
14	A/duck/Guangxi/413D40/2020(H9N2) AIV	GVRI	+	-	-	+	-	+	-	-	-	-	-	-	-
15	A/Duck/HK/77/76 (H2N3) AIV	UHK	+	-	-	-	-	-	+	-	-	-	-	-	-
16	A/Duck/HK/876/80 (H10N3) AIV	UHK	+	-	-	-	-	-	+	-	-	-	-	-	-
17	A/Turkey/Ontario/6118/68 (H8N4) AIV	UHK	+	-	-	-	-	-	-	+	-	-	-	-	-
18	A/Duck/HK/862/80 (H12N5) AIV	UHK	+	-	-	-	-	-	-	-	+	-	-	-	-
19	A/dove/Guangxi/288P43/2017(H3N6) AIV	GVRI	+	-	-	-	-	-	-	-	-	+	-	-	-
20	A/duck/Guangxi/LZ038E117/2019(H4N6) AIV	GVRI	+	-	-	-	-	-	-	-	-	+	-	-	-
21	A/duck/Guangxi/330D18/2018(H6N6) AIV	GVRI	+	-	-	-	-	-	-	-	-	+	-	-	-
22	A/dove/Guangxi/295D25/2017(H4N8) AIV	GVRI	+	-	-	-	-	-	-	-	-	-	-	+	-
23	A/duck/Guangxi/423D20/2020(H4N8) AIV	GVRI	+	-	-	-	-	-	-	-	-	-	-	+	-
24	A/goose/Guangxi/292G39/2017(H3N8) AIV	GVRI	+	-	-	-	-	-	-	-	-	-	-	+	-
25	A/Duck/PA/2099/12 (H11N9) AIV	UP	+	-	-	-	-	-	-	-	-	-	-	-	+
26	B/Guangxi/1418/15	GXCDC	-	-	-	-	-	-	-	-	-	-	-	-	-
27	B/Guangxi/1470/15	GXCDC	-	-	-	-	-	-	-	-	-	-	-	-	-
28	NDV F48	CIVDC	-	-	-	-	-	-	-	-	-	-	-	-	-
29	IBV M41	CIVDC	-	-	-	-	-	-	-	-	-	-	-	-	-
30	ILTV Beijing strain	CIVDC	-	-	-	-	-	-	-	-	-	-	-	-	-
31	ARV S1133	CIVDC	-	-	-	-	-	-	-	-	-	-	-	-	-
32	FAdV4-GX005	GVRI	-	-	-	-	-	-	-	-	-	-	-	-	-

GVRI, Guangxi Veterinary Research Institute, China;UHK, University of Hong Kong, China; UP, University of Pennsylvania, USA;UC, University of Connecticut, USA; CIVDC, China Institute of Veterinary Drug Control; GXCDC, Guangxi Provincial Center for Disease Control and Prevention, China.
